# Association of Race With Lung Cancer Risk Among Adults Undergoing Lung Cancer Screening

**DOI:** 10.1001/jamanetworkopen.2021.4509

**Published:** 2021-04-06

**Authors:** Christine S. Shusted, Nathaniel R. Evans, Hee-Soon Juon, Gregory C. Kane, Julie A. Barta

**Affiliations:** 1 The Jane and Leonard Korman Respiratory Institute, Division of Pulmonary and Critical Care Medicine, Department of Medicine, Sidney Kimmel Medical College at Thomas Jefferson University, Philadelphia, Pennsylvania; 2 The Jane and Leonard Korman Respiratory Institute, Division of Thoracic Surgery, Department of Surgery, Sidney Kimmel Medical College at Thomas Jefferson University, Philadelphia, Pennsylvania; 3 Division of Population Science, Department of Medical Oncology, Thomas Jefferson University, Philadelphia, Pennsylvania

## Abstract

This cross-sectional study examines whether race is associated with differences in 6-year lung cancer risk among patients eligible for US Preventive Services Task Force–recommended lung cancer screening.

## Introduction

African American individuals at high risk of lung cancer may experience greater mortality benefit from annual lung cancer screening (LCS) compared with White individuals.^1,2^ However, African American individuals develop lung cancer with fewer pack-years of smoking and at younger ages than White individuals, and they are less often eligible for LCS when using United States Preventive Services Task Force (USPSTF) criteria.^3^ One approach to mitigate disparities in LCS eligibility is to use lung cancer risk prediction models to identify the patients with the highest risk.^4^ The Prostate, Lung, Colorectal, and Ovarian Cancer Screening Trial modified logistic regression model for lung cancer prediction (PLCOm2012) may improve sensitivity in lung cancer detection, specifically among African American individuals, and potentially reduce underrepresentation in screening cohorts.^5^ This study aimed to identify differences in PLCOm2012 6-year lung cancer risk among USPSTF-eligible LCS patients.

## Methods

The Thomas Jefferson University institutional review board approved this cross-sectional study and granted a waiver of informed consent because of the minimal risk nature of the research. This study followed the Strengthening the Reporting of Observational Studies in Epidemiology (STROBE) reporting guideline.

The Jefferson LCS Program is a centralized program at an urban, academic medical center. Sociodemographic and clinical data from patients screened from January 2018 to September 2020 were extracted from the LCS Registry. This included lung cancer risk using the PLCOm2012 model, calculated at entry into LCS, and diagnosis of screen-detected lung cancer.^6^ After excluding patients who self-reported races other than African American or White, risk quartiles were generated using median PLCOm2012 risk values for the screening and cancer cohorts. Descriptive statistics, independent *t* tests, χ^2^ tests, and Mann-Whitney *U* tests were performed using a *P* < .05 significance threshold. Statistical analyses were 2-sided and conducted using SPSS statistical software version 26 (IBM Corp) from October 5 to November 11, 2020.

## Results

Among 1276 individuals in the screening cohort, the mean (SD) age was 64.25 (5.81) years, 545 (42.7%) were African American individuals, and 757 (59.3%) were female individuals (Table). There were significant differences in gender distribution (African American: 343 female individuals [62.9%] vs White: 414 female individuals [56.6%]; χ^2^_1_ = 5.137; *P* = .02), smoking history (African American: 348 currently smoking [63.9%] vs White: 358 currently smoking [49.0%]; χ^2^_1_ = 27.967; *P* < .001), education level (eg, African American: 243 high school graduates [44.6%] and 37 college graduates [6.8%] vs White: 287 high school graduates [39.3%] and 98 college graduates [13.4%]; χ^2^_6_ = 31.500; *P* < .001), and insurance (eg, African American beneficiaries: 192 Medicaid or dual eligible [35.2%] and 138 private only [25.3%] vs White beneficiaries: 134 Medicaid or dual eligible [18.3%] and 243 private only [33.2%]; χ^2^_5_ = 58.992; *P* < .001) among races. Among the 32 patients with screen-detected lung cancer in the cancer cohort, 14 (44%) were African American.

**Table.** Baseline Characteristics of African American and White Patients Undergoing Lung Cancer Screening

Characteristics	Patients, No. (%)		*P* value
**1276 Patients in full sample**	**African American (n = 545)**	**White (n = 731)**	
Age, mean (SD), y	64.09 (5.70)	64.37 (5.89)	.40
Gender			
Female	343 (62.9)	414 (56.6)	.02
Male	202 (37.1)	317 (43.4)	
Ethnicity			
Hispanic/Latinx	1 (0.2)	48 (6.6)	<.001
Smoking status			
Current	348 (63.9)	358 (49.0)	<.001
Former	197 (36.1)	373 (51.0)	
Pack-years, mean (SD), y	48.71 (20.56)	57.95 (27.51)	<.001
Cigarettes, mean (SD)	21.62 (8.45)	25.89 (11.79)	<.001
Smoking duration, mean (SD), y	45.25 (7.15)	44.81 (8.18)	.304
Time since quitting, mean (SD), y	5.16 (4.97)	6.69 (5.45)	.001
Time smoked, mean (SD), y	45.25 (7.15)	44.81 (8.18)	.30
Personal history of cancer	83 (15.6)	161 (22.6)	.002
Family history of lung cancer	123 (22.6)	167 (22.8)	.91
COPD	241 (44.2)	292 (39.9)	.13
Body mass index, mean (SD)^a^	30.01 (7.27)	29.11 (6.42)	.02
Education			
Less than high school diploma	77 (14.1)	85 (11.6)	<.001
High school graduate	243 (44.6)	287 (39.3)	
Post high school training	19 (3.5)	28 (3.8)	
Some college	113 (20.7)	119 (16.3)	
College graduate	37 (6.8)	98 (13.4)	
Post graduate/professional degree	19 (3.5)	60 (8.2)	
Unknown	37 (6.8)	54 (7.4)	
Insurance status			
Medicare only	174 (31.9)	240 (32.8)	<.001
Medicaid/dual eligible	192 (35.2)	134 (18.3)	
Private only	138 (25.3)	243 (33.2)	
Medicare with supplement	26 (4.8)	69 (9.4)	
Private with supplement	10 (1.8)	24 (3.3)	
Other/none	5 (0.9)	21 (2.9)	
**32 Patients with screen-detected lung cancer**			
**Characteristics**	**African American (n = 14)**	**White (n = 18)**	
Age, mean (SD)	64.86 (5.60)	66.44 (5.87)	NA
Gender			
Female	12 (85.7)	11 (61.1)	NA
Male	2 (14.3)	7 (38.9)	NA
Histology			NA
Adenocarcinoma	11 (78.6)	10 (55.6)	NA
Squamous cell carcinoma	1 (7.1)	3 (16.7)	NA
Small cell carcinoma	0 (0.0)	3 (16.7)	NA
Neuroendocrine carcinoma	0 (0.0)	1 (5.6)	NA
Non-small cell carcinoma	2 (14.3)	1 (5.6)	NA
Stage			
I	10 (71.4)	9 (50.0)	NA
II	1 (7.1)	0 (0.0)	NA
III	2 (14.3)	4 (22.2)	NA
IV	1 (7.1)	2 (11.1)	NA
Limited stage small cell	0	1 (5.6)	NA
Extensive stage small cell	0	2 (11.1)	NA

Abbreviation: COPD, chronic obstructive pulmonary disease.

^a^ Body mass index is calculated as weight in kilograms divided by height in meters squared.

In the screening cohort (median PLCOm2012 risk of lung cancer: 4.75%; interquartile range [IQR], 2.63%-8.47%), African American individuals had a significantly higher median risk of lung cancer compared with White individuals (median PLCOm2012 risk of lung cancer among African American individuals: 5.81%; IQR, 3.42%-9.79% vs White individuals: 4.10%; IQR, 2.14%-7.26%; *P* < .001) (Figure). When comparing among risk quartiles, 326 of African American individuals (59.8%) undergoing screening had a risk score in quartiles 3 and 4 compared with 311 White individuals (42.5%). However, in the cancer cohort (median PLCOm2012 risk: 7.23%; IQR, 2.37%-12.64%), White individuals had a higher median risk compared with African Americans (median PLCOm2012 risk of lung cancer among White individuals: 10.55%; IQR, 2.21%-16.16% vs African American individuals: 5.87%; IQR, 2.89%-7.88%; *P* = .18), but this result did not reach statistical significance. Moreover, 11 White individuals (61.1%) with screen-detected lung cancer had a risk score in quartiles 3 and 4, compared with just 5 African American individuals (35.7%).

**Figure.**
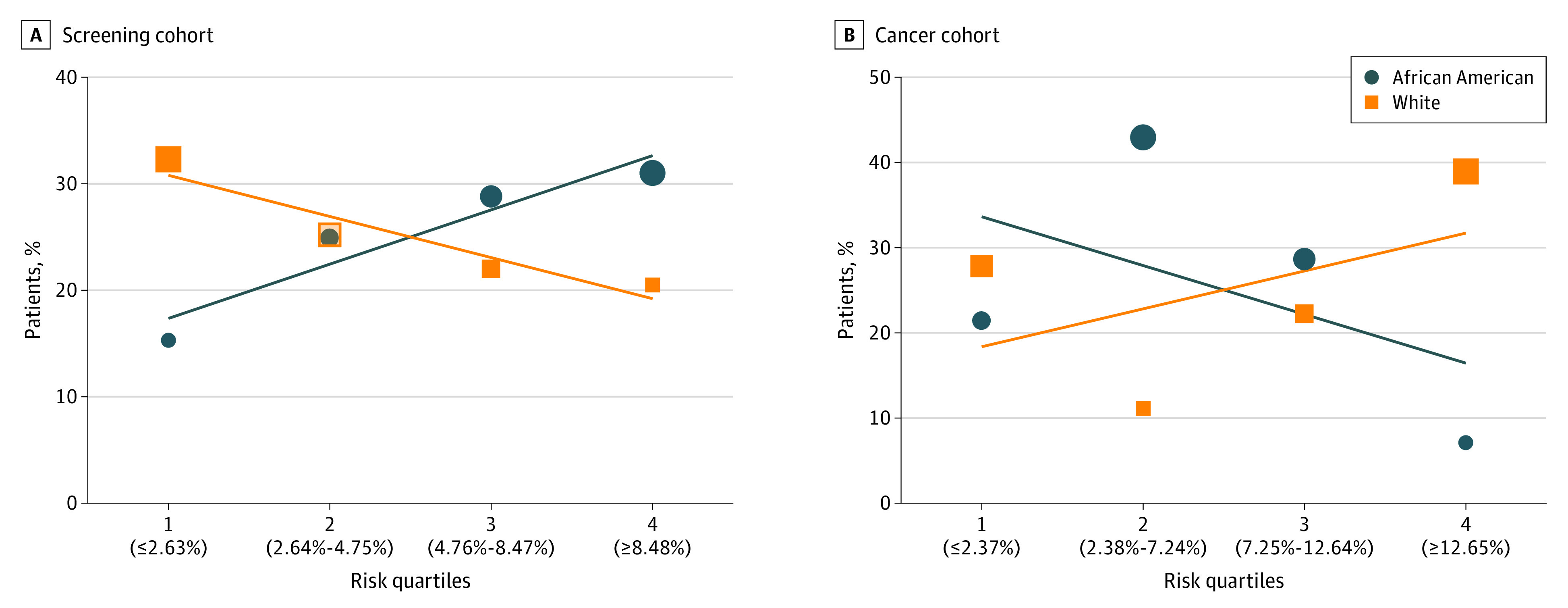
Six-Year Lung Cancer Risk Among the Screening and Cancer Cohorts by Risk Quartile A, Percentage of patients who underwent lung cancer screening in each risk quartile by race. African American and White individuals have overlapping percentages, with 24.9% and 25.2% of each cohort in risk quartile 2. B, Percentage of patients who had a screen-detected lung cancer in each risk quartile by race. The size of each data icon is proportional to the number of patients in each risk quartile. Risk quartiles are determined by median PLCOm2012 risk values (with cutoffs shown in parentheses along the x-axis).

## Discussion

To our knowledge, this study is the first to measure PLCOm2012 lung cancer risk among screened individuals and include more than 40% African American participants. We found that lung cancer risk scores were not aligned with lung cancer diagnoses in African American patients. In fact, African American individuals with screen-detected lung cancers were clustered predominantly in the lower risk quartiles. However, among White individuals undergoing screening, higher risk scores were associated with lung cancer diagnoses.

Our findings suggest that we should use caution in applying risk models to diverse populations, given that our current understanding of lung cancer risk is incomplete. Existing models have been derived from screening trials including 5% or fewer African American individuals and may not apply equitably to real-world screening participants.^2,4^ Although our study is limited by its single-center approach and short duration of follow-up, the racially diverse patient population allowed us to identify weaknesses in risk calculation. Determinants of health, including social constructs and environmental factors, may be critical moderators of lung cancer risk among underserved populations. Further research on comprehensive risk prediction for underrepresented racial and ethnic populations should prioritize diversity and focus on additional factors related to socioeconomic status, geographic variables, the environment, and exposure history.
